# Prioritising well‐being and resilience to ‘build back better’: insights from a Dominican small‐scale fishing community

**DOI:** 10.1111/disa.12541

**Published:** 2022-06-17

**Authors:** Johanna Forster, Clare Shelton, Carole S. White, Agathe Dupeyron, Alena Mizinova

**Affiliations:** ^1^ Associate Professor at the School of International Development and the Tyndall Centre for Climate Change University of East Anglia United Kingdom; ^2^ Lecturer at the School of International Development and the Tyndall Centre for Climate Change University of East Anglia United Kingdom; ^3^ Research Fellow at the School of International Development and the Tyndall Centre for Climate Change University of East Anglia United Kingdom; ^4^ PhD Researcher at the School of International Development University of East Anglia United Kingdom

**Keywords:** agency, Caribbean, disaster risk management, environmental hazards, small‐scale fisheries, social well‐being

## Abstract

Climate change is increasing the severity of extreme weather events, particularly hurricanes, presenting a significant challenge to Caribbean coastal communities. In the aftermath of a major disaster, government interventions typically prioritise infrastructure, assets, and the economy through rebuilding roads, reviving economic sectors, and providing financial compensation. This is driven by a focus on macro‐level quantitative indicators rather than by local, multidimensional subjective and relational factors, closer to lived experiences and livelihoods. Using frameworks outlining social well‐being and agency, this paper explores strategies used by a fisheries‐dependent community in Dominica to recover from Hurricane Maria in 2017 and pursue well‐being. The findings highlight the importance of multidimensional well‐being, particularly relational and subjective dimensions, including existing social networks, and personal relationships critical for recovery after Maria. Furthermore, the paper demonstrates how recovery initiatives that concentrate solely on material well‐being, such as employment, can undermine agency in the capacity of a community to recover and build resilience.

## Introduction

Despite the concept of ‘building back better’ becoming increasingly prominent in calls to build more resilient and sustainable communities to disaster risk, local voices and priorities are often not central to hazard response and recovery efforts (Mannakkara and Wilkinson, 2014; Collodi et al., 2021). Alongside the promises of the ‘building back better’ agenda, stemming from the Sendai Framework for Disaster Risk Reduction 2015–2030, there is growing recognition that other, more intangible indicators capturing lived experiences relevant at smaller subnational levels, such as well‐being and agency indicators, are critical components of interventions supporting recovery and future resilience to environmental hazards and climate change (see, for example, Imperiale and Vanclay, 2020; Prayag, Ozanne, and Spector, 2021). Well‐being constitutes a process and an outcome that includes material resources (such as from income and assets) and non‐material aspects comprised of the social, cultural, or psychological factors fundamental to how people live, and the ways in which they pursue their well‐being goals (White, 2010). In particular, the role of the intangible social and relational components of well‐being in facilitating or hindering post‐disaster recovery warrants greater attention (Prayag, Ozanne, and Spector, 2021). It is argued that in some cases, the intangible losses may be equal to or of greater significance than tangible losses for those affected by disasters (Alston, Hargreaves, and Hazeleger 2018).

Understanding multidimensional well‐being requires a deeper understanding of the complexity encompassed within lived experiences (White, 2010). For instance, national‐level government recovery processes typically focus on economic recovery or development at macroeconomic scales, with less focus on smaller businesses and local community livelihoods (Mannakkara and Wilkinson, 2014). Livelihoods operate at smaller scales and rely on complex social relationships; successful recovery of these livelihoods and relationships thus requires that governments interact meaningfully with local communities and civil society organisations (Jigyasu, 2012).

Complex livelihoods, particularly those dependent on natural resources that usually entail multiple activities such as fishing and agriculture, are often overlooked in recovery and assistance efforts (Wiles, Selvester, and Fidalgo, 2005). For example, following Hurricane Ivan in Grenada in September 2004, the Red Cross assisted small‐scale farmers with agricultural inputs, and farmers were able to take advantage of higher prices for agricultural goods to recoup losses and recover livelihoods. However, this effort centred on single rather than mixed livelihood strategies and not all of those in need were provided with support (IFRC, 2008).

Fisheries and fishery‐dependent communities, especially small‐scale fisheries in tropical latitudes, are considered to be particularly vulnerable to disasters triggered by natural hazards, fluctuating environmental conditions, and climate change (Coulthard, 2008; Naskar et al., 2021). This stems from being both physically vulnerable to the hazards themselves, as well as economically and socially vulnerable to, inter alia, impacts on or changes to fish stocks, resource access, and market systems that can then lead to poverty (Seara, Pollnac, and Jakubowski, 2020). For this reason, supporting fishers and their communities following a disruption or disaster must be informed by a fuller appreciation of complex livelihoods, specifically because mixed livelihood strategies are commonplace among fishers, who switch between fishing and other sources of income (such as construction, tourism, and agriculture) during the year when adverse weather or extreme events render fishing dangerous or impossible (Forster et al., 2014; Karlsson and Mclean, 2020). However, measures of well‐being that are further removed from what people do and why, encompassing the way a person feels, are less frequently derived from routinely collected data following a disruption or disaster (Morgan et al., 2015). For fishing communities, there is longstanding evidence of the importance of subjective well‐being. Being a fisher is a way of life, not just an occupation; fishers attain social and psychological well‐being by being able to fish (Pollnac, Pomeroy, and Harskes, 2001). Similarly, there is mounting evidence of the importance of having strong pre‐existing relational well‐being, derived through social networks and community cohesion and cooperation, and strong social capital, which influences a fishing community's ability to recover after a disaster (Marin et al., 2015; Gillam and Charles, 2018).

By analysing the intersections and interactions between these well‐being dimensions and how they relate to recovery and future resilience, we can improve our understanding of individual and collective actions and responses following hazard events. Strategies for responding to hazards are multiple and interconnected and can reflect resilience at different levels—and a key factor in social resilience is agency. Previous research has called for increased attention to be given to understanding adaptive strategies and resilience, and how they relate to the agency of those at risk (see, for example, Adger et al., 2008; McLaughlin and Dietz, 2008; Coulthard, 2012a). Agency has been referred to as the blind spot in social‐ecological systems resilience, and other frameworks can be useful in bridging this gap in understanding social resilience (Calderón‐Contreras and White, 2020). This is important for disaster recovery as there may be trade‐offs between strategies to improve resilience and individual well‐being, for instance: actions that may make an island more resilient by moving people away from vulnerable coastal areas may reduce individual well‐being if important social networks are disrupted.

This paper aims to explore, through a typology of agency (Lister, 2004), how response strategies are chosen following a disaster and how they affect social well‐being and resilience. We direct our inquiry to the Commonwealth of Dominica (hereafter Dominica), which experienced the devastating impacts of Hurricane Maria in September 2017, while still recovering from Tropical Storm Erika in August 2015. The study concentrates on three small fisheries‐dependent communities on the island's exposed east coast, providing an ideal context to explore multiple response strategies and what these mean for recovery and well‐being. This includes moving recovery beyond a focus on physical infrastructure and livelihoods to highlight the importance of social and relational elements.

## Approach and methods

### Exploring well‐being and agency in hurricane responses

**Figure 1:**
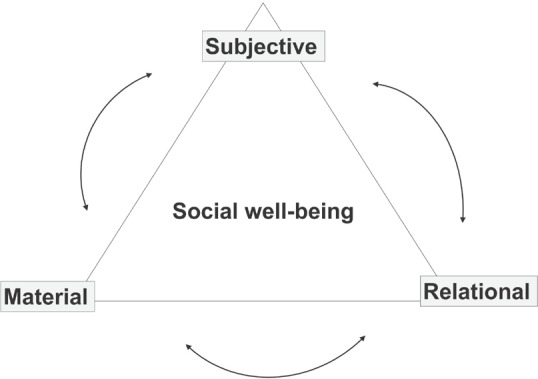
Social well‐being **Source**: authors, adapted from McGregor (2007) and White (2010).

We applied the multidimensional concept of well‐being (Gough and McGregor, 2007; White, 2010), also referred to as social well‐being, to understand how well‐being influences recovery and resilience and vice versa, to frame our inquiry and analysis of actions and responses following a disaster. Social well‐being has three interrelated dimensions, which exist within an ‘enabling environment’, providing the conditions and structures where well‐being is pursued. The three dimensions are: (i) a *material* dimension that considers the resources people have and the extent to which their human needs are met; (ii) a *relational* dimension to address the extent to which social relationships enable, or disable, the person in pursuing well‐being; and (iii) a *subjective* dimension that assesses their own level of satisfaction with the quality of life they achieve (see Figure 1) (McGregor, 2007; White, 2010).

The social well‐being approach considers both a person's objective circumstances, which may be measured quantitatively, and makes a subjective evaluation of one's satisfaction with quality of life, which can be measured qualitatively (Gough and McGregor, 2007). Social well‐being can be defined as ‘[a] state of being with others and the natural environment that arises where human needs are met, where individuals and groups can act meaningfully to pursue their goals, and where they are satisfied with their way of life’ (Armitage et al., 2012). This conception of well‐being recognises that living well is more than fulfilling basic survival needs, income, or happiness, and includes social and physiological needs constructed between an individual and society. This collective or ‘social’ conception of well‐being, where perceptions not only reflect individual preferences and aspirations, but also shared values and understandings of society that affect individual and collective choices driven by the pursuit of well‐being, is central to this approach (Gough and McGregor, 2007). The relational dimension recognises that material and subjective dimensions of human lives do not occur in a social vacuum; well‐being is dynamic and framed by complex social relations (Gough and McGregor, 2007; McGregor, McKay, and Velazco, 2007).

Strong relational well‐being can increase community resilience (Gillam and Charles, 2018) in a disaster context, where this refers to the community responding to change by drawing on communal resources, to overcome adversity and take advantage of new opportunities (Berkes and Ross, 2013). This focus on community resources emphasises the collective nature of adapting to change, in contrast to household‐ or individual‐level approaches to social resilience.

Social networks are recognised as important before, during, and after disasters (see, for example, Akbar and Aldrich, 2018; Carstensen, Mudhar, and Munksgaard, 2021). The multidimensional well‐being framework provided the structure in our inquiry to explore not only material assets lost or damaged, as is typical in a post‐disaster needs assessment (PDNA) (CoD, 2017), but also these critical subjective and relational dimensions of well‐being and the context in which they were affected or changed following Hurricane Maria. However, we also recognise that tensions can arise due to cultural and social practices. In particular, power associated with relationships (at differing levels) can hinder people's agency and cause tensions. These tensions have the capacity to strip individuals and communities of personal choice and can lead to power differentials, exploitation, or the exacerbation of poverty (McGregor, 2007). This is where considering the concept of agency is especially important in understanding social well‐being.

**Figure 2:**
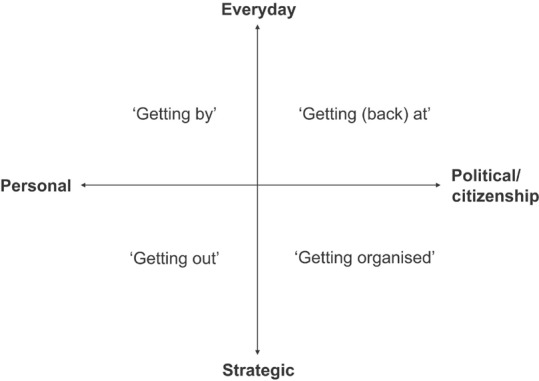
Forms of agency exercised by people in poverty **Source**: Lister (2004, p. 130).

The way agency is expressed is inextricably shaped by social factors, such as values, risk perceptions, and culture, which influence who is able to make decisions, and how (Lister, 2004; Adger et al., 2008). How people exercise their agency following a disaster is influenced not only by these factors, but also by how people perceive and pursue their well‐being, that is, their aspirations and constructions of well‐being. As Deneulin and McGregor (2010) note, the role of power in determining the extent to which well‐being can be pursued highlights the need to understand people's agency. We unpack different expressions of agency using Lister's (2004) framework (see Figure 2), examining the types of strategies people took in the immediate aftermath and the subsequent two years after Hurricane Maria. While originally developed to appraise people's responses to poverty, Lister's agency framework has since been adapted and applied to a fisheries context (Coulthard, 2012a, 2012b; White, 2015) to understand better how people respond to changes in their social and physical environment.

The four types of strategies outlined in Figure 2 refer to different levels of agency, including from the personal, reflecting individual choices, to the political/citizenship, related to the capacity of people to effect wider change. Whether a strategy is considered as ‘everyday’ or ‘strategic’ often reflects whether actions are aimed at addressing short‐term issues or whether they are concerned with longer‐term strategic goals (Lister, 2004). Lister (2004) describes the four categories as *‘getting by’, ‘getting out’, ‘getting (back) at’*, and *‘getting organised’*.

‘Getting by’ refers to actions such as continuing to engage in current livelihoods, diversifying livelihoods (such as by adding value to agricultural or fishery products), and changing preferences to meet conditions better. Coping strategies and ‘getting by’ are often used interchangeably, and ‘getting by’ can also be thought of as one example of coping (Lister, 2004). Coping broadly refers to how people act within the limits of the resources upon which they draw and their own expectations of what is needed, which can include well‐being (Wisner et al., 2004). Coping strategies and capacities are commonly discussed in the context of hazards and resilience as the ways in which people deal with hazards and their aftereffects, and their capacity to anticipate and respond to a hazard (Wisner et al., 2004; Tierney, 2014). ‘Getting out’ refers to actions and strategies that are more focused on long‐term strategic goals, such as migration or changing livelihoods altogether. ‘Getting (back) at’ encapsulates forms of resistance and can include circumventing formal institutions and structures or engaging in illegal activities for income. Finally, ‘getting organised’ is agency exercised to effect change, such as collective action to obtain access or property rights or to mobilise civil society. Many of these actions, especially the more strategic, rely on social networks and personal connections, either to find new employment and income opportunities or to engage collectively at political levels (Lister, 2004; Coulthard, 2012a). The framework dimensions of ‘everyday–strategic’ and ‘personal–political/citizenship’ are considered as continua rather than dichotomies, and any one individual may be exercising all four forms of agency in their life at a given time (Lister, 2004). While coping strategies are frequently discussed in the literature on disasters (see Wiser et al., 2004), using the Lister (2004) framework helps to elucidate the motivations for specific strategies and the way these are shaped by available resources and driven by goals for achieving well‐being.

### Dominica

Dominica is an island nation in the Caribbean Sea with a population of slightly more than 71,000. Its landmass of around 750 square kilometres is surrounded by approximately 180 kilometres of coastline (Pinnegar et al., 2019). On land, volcanic peaks create a precipitous central mountain range, and steep topography (Barclay et al., 2019). Seagrass, mangrove, and coral reef habitats are found along the coast, but they tend not to be particularly extensive. The most productive coral colonies and associated fisheries are found within 250 metres of the shore (Pinnegar et al., 2019).

Dependence on fishing for consumption and income is relatively high in Dominica, as compared with global averages (20.5 kilograms), with fish consumption at 27.1 kilograms/per year/per capita (FAO, 2018, 2020). Records show that in 2017, 912 people (17 women) were engaged in fishing in Dominica (FAO, 2018), operating from 31 ports/landing sites (including undesignated sites) (Fisheries Division, Government of the Commonwealth of Dominica, 2012). The majority (23) of these are situated on the west coast, with two in the south and six on the east coast (Fisheries Division, Government of the Commonwealth of Dominica, 2012). It is estimated that the sector employs some 2,200 people overall (CoD, 2017). Fisheries mainly target pelagic species, such as tuna, marlin, and dolphinfish through the use of fish aggregating devices (FADs), first introduced in 1987 to improve catches of large migratory species (Theophille, 2016). There are also minor reef‐related and demersal fisheries that use traps and nets (Pinnegar et al., 2019). Fishing in Dominica is described as small‐scale and artisanal, and often for subsistence purposes. According to the *Fisheries Industry Census of Dominica 2011*, many fishers fulfil multiple roles in the industry, such as gear or boat builders/repairers and outboard engine mechanics, while some also act as vendors. The local vessels are small open boats, powered by at least one outboard engine and usually operated by two fishers (Fisheries Division, Government of the Commonwealth of Dominica, 2012).

Dominica's climate is characterised as tropical maritime, with dry (December–May) and rainy (June–November) seasons. Hurricanes can occur at any time during the rainy season (with August and September being the peak months) (World Bank Group, 2022). The most recent major hazard to affect the island was Hurricane Maria, a Category 5 event on 18 September 2017 (CoD, 2017). The storm caused catastrophic destruction across the island, affecting 80 per cent of the population and destroying or damaging more than 90 per cent of the buildings and infrastructure. According to the PDNA, 30 people lost their lives (CoD, 2017) and 37 people are still declared missing,^2^ and thousands more were injured owing to flooding and landslides (Schnitter et al., 2019). Tropical Storm Erika preceded Maria by two years, and despite being categorised as a less intense storm, resulted in large loss of life (14 confirmed fatalities and 17 people missing, plus more than 500 rendered homeless) and substantial impacts on the island's infrastructure and natural environment (Heron, 2018; Barclay et al., 2019).^3^ Based on an assessment of impacts across sectors, including agriculture, fisheries, forestry, and tourism, the Government of Dominica's PDNA concluded that Hurricane Maria resulted in total damage of USD 931 million and losses of USD 382 million, amounting to 226 per cent of gross domestic product (GDP), for 2016 (CoD, 2017). The fisheries sector was estimated to have suffered combined damage and losses of USD 3 million, and flagged as particularly vulnerable to future hurricane activity due to myriad consequences of climate change (Pinnegar et al., 2019). In response to the devastation wrought by Maria, the Government of Dominica committed to becoming the ‘world's first climate resilient nation’ (CoD, 2020b, p. 13), through the adoption of ambitious strategic initiatives across all sectors to ‘build back better’.

#### Case study context: San Sauveur, Petit Soufrière, and Good Hope

**Figure 3:**
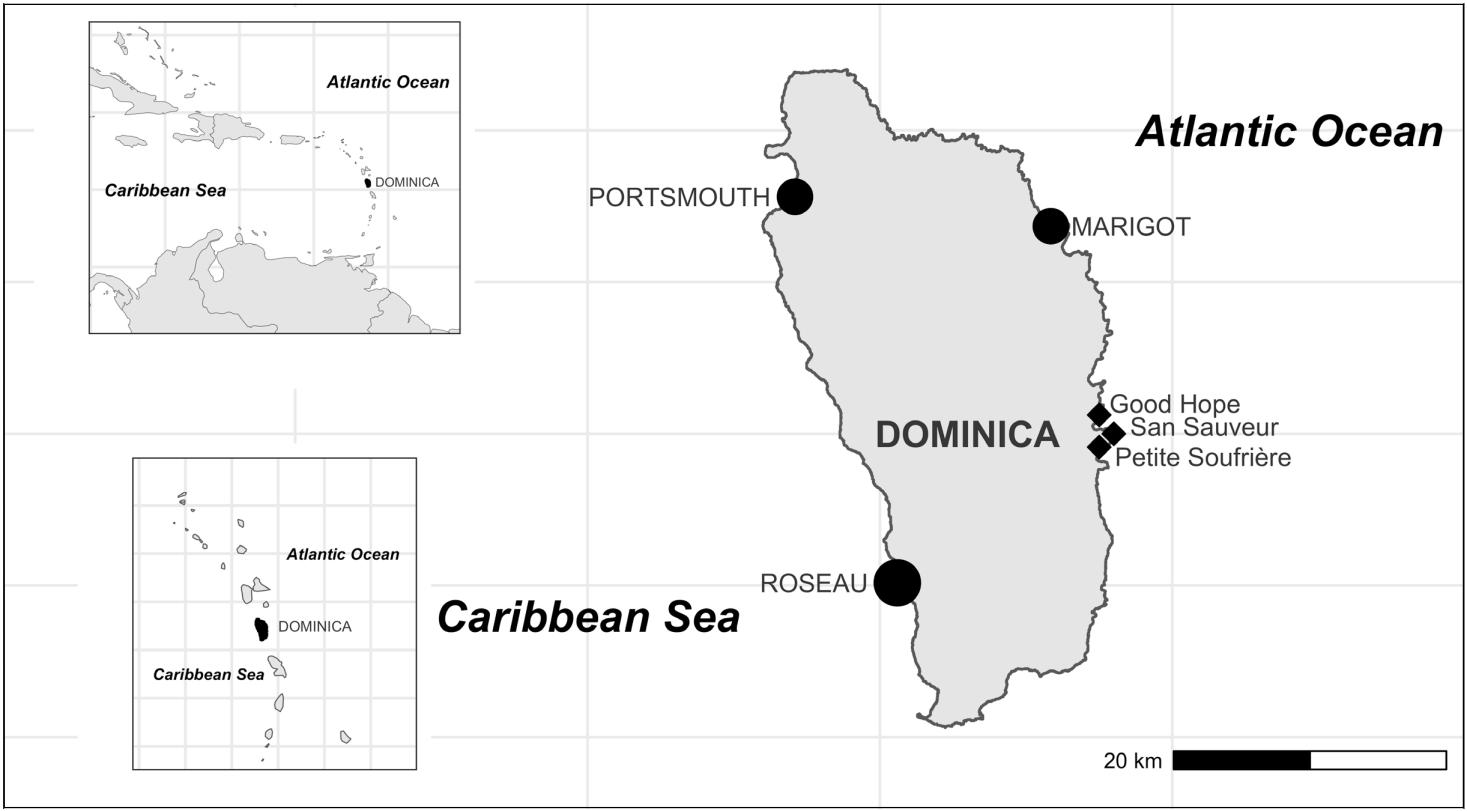
Map of Dominica in the eastern Caribbean∗ **Note**: ∗ The map shows the location of the three study site communities (diamonds), the capital, Roseau (large circle), and two major fish landing sites (medium circles). **Source**: authors.

The village of San Sauveur in Saint David Parish, on the exposed east coast, is the primary focus of this study, together with the twinned villages of Petit Soufrière and Good Hope, collectively hosting a population of around 800 (see Figure 3). There are approximately 10 fishing boats that regularly use the San Sauveur landing site, and in most cases, they are operated by two fishers—there are an estimated 20 fishers in this locality (Fisheries Division, Government of the Commonwealth of Dominica, 2016). These villages were severely impacted by Hurricane Maria: roads were damaged or made impassable, leading to transport and communications links being cut off from neighbouring villages, larger towns, and the capital, Roseau. The local agricultural sector surrounding these villages was devastated, with wind damage affecting banana and bay crops, and landslides and flooding leading to the loss of mainstay ground provisions, dasheen and cassava. As a result, the fisheries sector emerged as particularly important to recovery efforts in the immediate aftermath of the hurricane, through local provision, and delivery by sea, of emergency water and food (Turner, McConney, and Monnereau, 2020).

#### Data collection and analysis

Fieldwork was conducted during October 2019, two years after Hurricane Maria, with the aim of providing a snapshot (rather than a longitudinal assessment) of post‐disaster recovery responses. The methodology allowed us to explore immediate and short‐term responses and the strategies people employed in the medium term (more than one year) that reflect more strategic expressions of agency, and how they affected social well‐being. Meetings with national‐level key informants together with community profiling served to amass contextual information and enabled a ‘snowball sampling’ approach for further interviews (Coulthard et al., 2015). These initial scoping activities allowed us to paint a picture of the community structure, initiated our snowball sampling method (whereby respondents recommended other potential interviewees), and provided us with a list of invitees to the initial community meeting. This meeting was held in San Sauveur village on 9 October 2019 with 25 participants and offered the opportunity to meet people living and working in the case study villages (see Figure 3), discuss the study aims, and learn about major changes and events that have affected the communities.

Semi‐structured interviews at the community (n=12) and national (n=10) level (see Table 1), as well as three community focus groups with between two and five participants each (Village Council (two), Parish Fisheries Cooperative (two), and a local youth group (five)) were held to garner a wide range of perspectives. At the community level, interviews and focus groups took place at fish landing sites, in communal areas, or in people's homes, with individuals involved in the fishing industry (past and present) or prominent members of the community (such as a health professional and school principal). The fishers who took part in this study ranged from persons who had worked as such all of their lives and continued to do so in a full‐time capacity, to those who worked part‐time and supplemented their income with other activities, such as farming or construction. Some fishers owned their boat and either fished alone or with friends and family members, whereas others shared the ownership of their boat and worked together in crews. We interviewed several women in the fishing community, including individuals who participated directly in fishing and those who sold/marketed fish at the landing site. The community interviews and focus groups included questions to explore what is meant by recovery and how hurricane impacts affected different dimensions of well‐being (material, subjective, and relational). The questions facilitated discussions to determine the important relationships within the community and externally, how people feel, the choices they make, and why they make them, with respect to their lives and livelihoods, their homes, and the wider community infrastructure. We also asked questions about future aspirations for individuals and for the community.

**Table 1:** Details of community‐ and national‐level interviews∗

	Community	National
Department of Fisheries (3)		✓
Bureau of Gender Affairs (1)		✓
Department of Local Government and Community Development (1)		✓
Climate Resilience Execution Agency for Dominica (1)		✓
Red Cross (1)		✓
Community development officers (2)		✓
Academic historian (1)		✓
Health professional (1)	✓	
School principal (1)	✓	
Parish Fisheries Cooperative (2)	✓	
Fishers/ex‐fisher (6 total: 2 women, 4 men)	✓	
Fish vendor (1)	✓	
Hospitality/coastal tourism (1)	✓	
**Total**	**12**	**10**

**Note**: ∗ The numbers in parentheses denote separate individuals.

**Source**: authors.

At the national level, interviews involved representatives of relevant government departments (see Table 1), plus other key national‐level stakeholders. These interviews provided the opportunity to garner information on wider factors and at varying scales that relate to post‐hurricane recovery, such as policies or initiatives on community development, disaster risk management, and fisheries management, and country‐level economic, social, and cultural information to contextualise further the community‐level data. Table 1 contains a summary of participants who contributed to the study.

All interviews and focus groups were undertaken in English, and the majority were recorded and transcribed verbatim (lasting between 30 and 90 minutes); where recordings were not possible detailed notes were made. Transcriptions were coded in NVivo 12. The coding used both a deductive and inductive approach: codes were generated deductively in the first phase, informed by the social well‐being and agency frameworks described earlier to assign response categories for coded text. Phase two explored the data inductively, allowing for a more explorative approach and the opening up of issues and concerns, and analysis of where responses cross‐cut issues of recovery, well‐being, and agency—a critical analytical step to enable identification of a broader and more complex array of responses. Trends and emerging response themes are presented in the analysis using Lister's (2004) four categories of agency detailed above, offering a structure to capture the complexity described by the qualitative data.

## Results: agency and well‐being after Hurricane Maria

**Figure 4:**
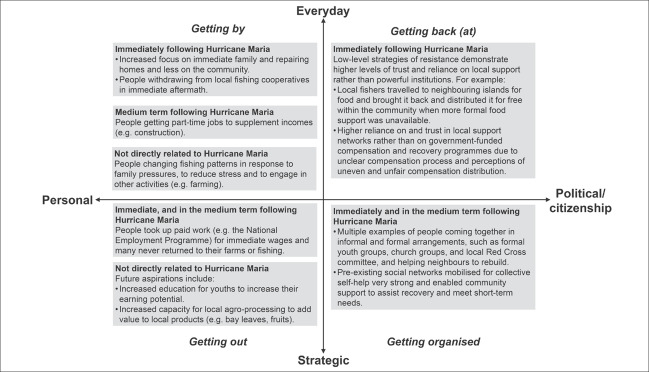
Forms of agency exercised in coastal Dominica after Hurricane Maria **Source**: authors, adapted from Lister (2004).

People's strategies and responses in the immediate aftermath and longer term following Hurricane Maria demonstrate nuanced reactions to the event itself (namely, the destruction of homes, loss of livelihoods, and the trauma of the occurrence), as well as efforts to support emergency relief and recovery by charities, aid agencies, and the government. The forms of agency expressed by participants are summarised in Figure 4 and discussed in the subsection below.

### Forms of agency

#### ‘Getting by’

‘Getting by’ can easily be taken for granted, but it is an active process that involves complex use of various resources (also referred to as ‘coping‘), such as social, cultural, material, and personal resources. Differing quantities and/or accessibility of these resources shape an individual's ability to get by. In the aftermath of Hurricane Maria, participants reported a variety of strategies that enabled them to cope, drawing on various resources to employ these strategies.

For example, many participants stated that people shifted their focus from the wider community to their immediate family and homelife. This included a strong focus on rebuilding their own homes and ensuring that they and their family were able to eat, rather than participating in collective entities such as the fishing cooperative or other groups. Other strategies after Maria involved rebuilding homes using different techniques and resources, such as using tiles and screws rather than nails and galvanised tin as roofing materials. Participants reported more people going into part‐time work to supplement incomes (including construction) and seeking employment away from the villages as a reaction to income loss. The permanency of this shift for some individuals straddles the line between ‘getting by’ and ‘getting out’. This represents a trade‐off between different dimensions of well‐being, whereby certain areas of well‐being are prioritised at the expense of others (such as income generation to rebuild the family home over community activities).

Participants reported noticing more people leaving the villages in search of other non‐fishing or farming employment post Maria, but this phenomenon began prior to the hurricane. Government worker A said: ‘You find everybody is getting like a part‐time job, like the grocery stores in town, like the supermarket. [Before] [t]hey wouldn't go to get part‐time jobs, they would come back to do fishing with their father’. Participants commonly referenced livelihood changes after Maria, however, and many of these are in relation to the National Employment Programme (NEP), a government‐funded training initiative established in 2013 to assist people with obtaining training or jobs.^4^ Many people used the NEP as a safety net in the immediate wake of Maria, but it was never intended as a long‐term employment scheme. This change in livelihoods, with people seeking part‐time paid employment, reflects wider changes in society, such as shifting to a more cash‐based economy and different levels of educational attainment by younger generations. As stated, these wider changes began prior to Maria and the NEP, but the shock of the disaster, combined with the opportunities presented by the NEP, appear to have accelerated developments.

The experience of Maria meant that the strategies and resources upon which people relied previously had to change. In the words of Fisher A: ‘It's different because everybody had to find something different to do, because it is not the same life we used to have’. Hurricane Maria marked a changing point in the lives and livelihoods of many participants, owing to the monumental impact that it had on individuals, the community, and the country. In addition to physical changes in the landscape and seascape, the social and livelihood terrain changed, too. Participants discussed drawing on personal resources such as creativity and flexibility (see also Turner, McConney, and Monnereau, 2020), as well as social resources such as friends and family networks, to diversify their livelihoods and maintain their day‐to‐day functions. While some of these strategies concentrated on the personal (such as the individual and the immediate family) and everyday survival, others demonstrated more strategic and longer‐term expressions of agency. Individuals and households engaged in multiple strategies simultaneously.

#### ‘Getting out’

‘Getting out’ refers to someone working consciously to change their livelihood and/or to exit fishing (or mixed agriculture and fishing). In the context of our study, we also use ‘getting out’ to refer to aspirations to improve livelihoods substantially, meaning that the livelihoods category may not change but there is a significant difference in how that strategy is pursued to improve income‐earning potential, such as via education for individuals, or their children. When livelihood strategies change (such as exiting a fishery), the transitions between different livelihoods can be complicated, particularly if paid work is insecure or incomes are volatile (Lister, 2004). This can mean that while people are ‘getting out’ of their coastal livelihood, these strategies may not always be aiding them in achieving their well‐being goals (see the ‘well‐being’ subsection below).

Participants reported short‐term responses to Maria that involved both ‘getting out’ of fishing and of the community itself. One of the reasons for this was the lack of appropriate compensation from government sources. The fish vendor remarked that: ‘There was government intervention where they provided the farmers with a stipend or some financial assistance monitoring, but that in itself did not do anything much, because […] those crops are crops that take anywhere between nine months to pick up again. So, it's like, you have to wait nine months to start to make some money’. This lack of support over the period it takes for crops to grow was also mentioned as a reason for people pursuing employment elsewhere, especially with the NEP—the NEP budget was doubled following Maria to allow it to absorb additional unemployed people (Beazley, 2018). However, in the two years since Maria, many individuals who left the study villages with jobs supplied by the NEP have not returned. Fisher B said: ‘When they implemented it, it was a good thing. Because then, just after Maria, everyone's out of work and everyone wants to make something, it was a good thing. They probably intended people to go back, to their farms and all that, after we recovered. Most people have not’. While initial NEP employment take‐up may well have been a coping action, the choice to stay away and ‘get out’ of original livelihoods was a strategic move for some individuals.

Aspirations for the future, to improve the local economy and one's earning potential, were also discussed. Participants talked about hopes for education and more generally for the community in terms of ways of ‘getting out’ of fishing, as well as providing very specific examples of actions to enhance local earning potential while maintaining mixed fishing and agricultural livelihoods. Many participants expressed a desire for the community to promote educational attainment as a mechanism to improve earning potential. As the health professional put it, ‘I would like to see the [younger] people go to college, and then come home and do some work here. Because most of them get the good jobs and they have to go and live there, and go to the town. So, I would like to see, if we have some factories or some things here, so we can have more persons employed in the area’. These education goals were related as ways to improve earning potential within the community, while maintaining current livelihoods (and existing social networks).

However, other participants described wanting different livelihoods for their children. The fish vendor underlined: ‘I would like to see persons engaged in reasonable professions, reasonable jobs, that can generate decent money and then be comfortable in their lives. … I would like my children to be more educated than I am, a lot of parents are like that’. Two participants mentioned a specific strategy in this regard: building agro‐processing plants (for bay oil or local fruits) to add value to locally grown produce and diversify community employment opportunities. Not all participants, though, have the same aspirations for the community: some envisaged a place where future generations could engage in the same livelihoods but earn better incomes, whereas others wanted their children to ‘get out’ of fishing and farming altogether.

#### ‘Getting (back) at’

‘Getting (back) at’ in this context includes behaviour that includes everyday challenges or acts of resistance to power structures. Everyday acts of resistance offer individuals a chance to repel the constraints placed upon them and ‘get (back) at’ those in power (Lister, 2004). While a number of participants reported that the government did not help enough in getting everyone back on their feet, not all of them shared that view.

Some participants seemed resigned to the situation, reporting both an acceptance that at least the government gave some support, as well as expressing frustration at the opaque decision‐making concerning compensation. Fisher C observed: ‘some people get very little. Like it wouldn't even cover a quarter of what they lost. And then again, at least they get back something’. At the same time, other participants felt that a lack of political connections, or being affiliated to the party in power, was a barrier to accessing resources. Fisher D emphasised: ‘Right now, if you're not in politics, you won't get nowhere you have to’.

Participants shared concerns about the lack of transparency in compensation decisions and what was viewed by several of them as unequal or puzzling amounts distributed in the wake of Hurricane Maria. For example, one full‐time fisher with several decades of experience reported getting one‐third of the compensation of other part‐time fishers who had only been fishing for relatively short periods. Participants reported that personal connections (such as knowing the individual liaising on compensation claims) were some of the major factors affecting whether compensation was received, and how much. Other participants more familiar with the administration of the compensation programme described how political motivations were less at play than an overall lack of organisation or centralised planning, meaning that compensation was handed out in an ad hoc fashion. Nevertheless, the result that many local people felt they could not trust in the programme may have contributed to a sense that they could not fully trust in the government and those in power to take care of them post Maria, leading to behaviours that circumvented formal institutions for the purpose of recovery.

Prior to Maria, strong local networks supported community members and were, and continue to be vital (see the ‘well‐being’ subsection below). These networks were key to people in the immediate aftermath and in the medium term following the hurricane. Local fishers with intact boats travelled to neighbouring islands and brought back food—often for free—to distribute within the community. Fisher E recalled that: ‘A lot of boats went to Guadeloupe, to Martinique—for food stuff. … Even for free. … Boats can just go … get stuff and then come back to their villages’. They did this rather than waiting for official assistance from either the government or external aid agencies, demonstrating higher trust in their community and their own agency as opposed to the central authority. These strategies, combined with the attitudes towards compensation above, reveal that at varying levels, a high value is placed on self‐ and community‐reliance and independence, as well as a lack of trust in the government or those in more powerful positions providing support. Furthermore, a key informant reported that the constituency of San Sauveur is marginal in terms of political allegiances, and so, even before the recovery efforts and compensation, trust may have been an issue among a large portion of the community. Taking care of oneself and the community, therefore, not only illustrates that these local community identities resonate more strongly than anything aligned with the government, but it also shows evidence of agency, expressed as subtle resistance. While there was no direct challenge to, or desire to challenge, the government or those in power, trust in formal structures set up by the latter is superseded by trust in local communities.

#### ‘Getting organised’

‘Getting organised’ here refers to collective self‐help and political action. Collective self‐help is an important expression of collective agency and can sometimes serve as a starting point for political action (Lister, 2004). Participants reported multiple examples of people coming together in both formal and informal ways to support each other. Traditionally in Dominica, this expression of coming together to help each other is known as *‘koudmen’*, a Creole word derived from the French ‘coup de main’ (Macfarlan, Quinlan, and Remiker, 2013), and it has been described as the ‘social glue’ that keeps communities together. Understandably, *koudmen* is prominent when there is a disaster, as it shapes how community members support each other in building back lives and livelihoods.

Formal examples of collective action included local youth groups that undertook activities for the benefit of the community, such as cleaning beaches, as well as the endeavours of the local Red Cross and church‐based entities. Numerous cases came to light of collective actions that demonstrated the strength of local relationships and networks (such as sharing resources and human capacity), to work collectively and individually to support individuals in need, in the immediate aftermath of Maria and in the subsequent months and years. The health professional noted: ‘important for community, is that everybody gets involved. You see, even if you're not talking to me, something happens and you will be around to come, that is the thing about our community, yes. It's small, but we have everybody involved in whatever. If there is a little accident or a little landslide, everybody comes together’. Coming together collectively to support each other *(koudmen)* continues to be an important aspect of community in Dominica after Maria.

### Well‐being

Social relationships at multiple levels (that is, between participants and another individual in the household, the community, or even at the national level) emerged as significant in understanding how one achieves ‘a good life’. People repeatedly mentioned the importance of family, friends, neighbours, crew, other fishers, respected members of the community, the church, and other prominent community groups, in terms of being instrumental in their ability to cope and to recover following Maria. These social relationships (encompassed by the relational well‐being dimension) have emerged as powerful means of dealing with and living through the trauma of a hurricane, providing resources to bolster social resilience (Heron, 2018). Fisher B expressed this succinctly: ‘For any community to thrive properly, they need to be able to interact with one another’. The value of strong social networks and self‐organisation leading to enhanced resilience led to some communities being described as ‘a nation within a nation’, as they relied on critical transboundary relationships with communities on other nearby islands for recovery support (see also Turner, McConney, and Monnereau, 2020). There were examples of people coming together informally, expressions of trust and unity within the community to help one another after Maria, and the consequence of this for a person's general sense of well‐being. Fisher B stated: ‘Someone's doing well, isn't really based on what they have. … It's how you treat other people in the community. … You see, here to help out, develop their place, develop their self’. These informal networks are vital for community functioning, as Fisher C described: ‘What we usually do is if we see someone who is in need of something. Okay, such as a fisherman, I've got the fish, I know that this person hasn't got money to buy the fish, and I see that they need it, I will give them’. These statements demonstrate not only the importance of social relationships, but also how participating as a community member is key to how a person lives a ‘good life’ and connects with the Dominican tradition of *koudmen*.

As well as informal relationships, participants also referred to more formal arrangements, ranging from community youth groups to the local Red Cross chapter to government organisations such as Parish Councils. Notwithstanding the importance of these wider local and national support networks, it was noticeable during the interviews that they were mentioned less frequently than the local social relationships that aided recovery following the hurricane.

What it means to be well for participants, or to have good subjective well‐being, encompassed an array of values and emotions, such as feeling grateful, comfortable, healthy, and happy, not feeling lonely, and having independence and enjoying choices in how to live life. However, specifically in relation to Maria, several participants underlined changes in subjective well‐being due to a renewed, or new, fear of hurricanes. A member of a local youth group said: ‘now that we're in the hurricane season, so you'll be thinking like “anytime [a] hurricane can come and you can lose everything again”. But like before you'd be like “okay, it's hurricane season”. They would say to be careful, okay, they would say the hurricane is coming, but in your mind, you'd be like “that might pass”’. This ties in with several other comments that were made describing a wider feeling in the community of fear and worry after Maria, and an associated decline in the mental health of some people, both young and old. A member of a Village Council remarked: ‘Every time we hear a storm coming, we get shaky … and we're traumatised, we never really got like, counselling for that … my mind, my mental state isn't the same anymore. I'm scared of everything now'. The importance of exercising agency was highlighted many times during these discussions, given the realisation that ‘anything can happen at any time’ and thus the assertion that you always need to be prepared.

The ability to prepare, and having financial independence, was commonly referred to in terms of material well‐being factors, but also reflects the values that participants deem crucial for achieving a ‘good life’, including having the financial assets and ability to bounce back after a hurricane. People commonly referred to having a range of high value assets (such as a house or vehicle), as well as employment, and significantly, not needing to rely on friends and family for financial assistance.

For participants engaged in fishing‐related activities, the loss of and damage to boats and gear (including FADs) post Maria were a major factor in people deciding to leave the sector and take up alternative employment. More indirect issues also contributed, such as a lack of electricity for storing fish, meaning that for up to seven months after Maria, fish vendors could only buy what they could sell that day, and fishers were not guaranteed to sell their catch. Compounding this issue was the loss of the largest fisheries complex on the island (in Roseau), which suffered extensive damage and has not to date (as of February 2022) been rebuilt to full capacity. Fisher A explained: ‘most of my fish used to go to the complex in Roseau … since Maria it was destroyed, and they haven't fixed it back. So that put a strain on us, we have to look for other customers to buy the fish from us’.

#### Top‐down aid and recovery efforts and initiatives

Critical to recovery efforts in these communities, and serving as a lens through which wider lessons for Dominica can be learned, is the interplay between individual strategic agency, how personal choices can affect well‐being, and the decisions and actions of the government and aid organisations. The first relief activities carried out by the government and the international community included the provision of in‐kind support (food, water, and non‐food supplies) (Beazley, 2018). It is not the purpose of this study to critique the social protection response to Hurricane Maria, but it would be remiss not to mention here the relevance of applying a social well‐being and agency lens to the response and recovery actions of national and international aid.

At the country level, it became clear that the NEP had a big influence on individual actions and responses following Maria. This programme provided much needed immediate financial support, yet, in some cases, it undermined personal and collective agency and well‐being, through multiple outcomes. These include declines in physical health and well‐being due to increases in the illnesses typically associated with poor diet and nutrition, such as hypertension and diabetes, because NEP assistance directed employment away from farms, so healthy fruit and vegetables are now harder to buy locally. Several other participants also noted that because people have had to work further away from home, farming and fishing livelihoods have often been abandoned completely. As one local academic familiar with the NEP observed: ‘wages are so low that the families in the country villages had to send food to the children who were working in this factory, and therefore it was not actually creating income at all’.

Movement of people out of the community for employment has led to changes in the social structure of the community (such as younger people leaving to seek work, or fishing and farming livelihoods changing as people became more reliant on the NEP) and has undermined vital relational well‐being—people clearly articulated the importance of community cohesion and social ties (such as also through *koudmen)* as features of how they define ‘living well’. For some, this programme has not only impacted on physical and mental health (subjective and relational well‐being), but years after Maria, people are also not necessarily more financially stable because of it either (material well‐being).

## Discussion: resilience and well‐being following Hurricane Maria

The challenges of building resilience to future environmental hazards and climate‐related disasters have been recognised nationally and regionally across governments, research entities, and development organisations in the Caribbean. Against the backdrop of Hurricane Maria, the Government of Dominica commenced its attempts to transform the island into the ‘world's first climate resilient nation’ (CoD, 2020b, p. 13), by establishing the Climate Resilience Act (CoD, 2018), providing the mandate to create the Climate Resilience Execution Agency for Dominica (CREAD), set up in 2018 (with a four‐year mandate until 2022). Drawing on the National Resilience Development Strategy: Dominica 2030 (NRDS), which outlines the policy framework to guide the island's recovery (CoD, 2020a), the Dominica Climate Resilience and Recovery Plan 2020–2030 (CRRP) (CoD, 2020b) was developed, under the leadership of CREAD, and published in 2020. The NRDS stipulates that the CRRP should reflect three ‘pillars of resilience’: (i) climate resilience systems; (ii) prudent disaster risk management systems; and (iii) effective disaster response and recovery—across key target areas that include support for communities, the economy, infrastructure, and institutional systems.

Specifically, for Caribbean coastal communities and the fisheries sector, various large‐scale initiatives have also been established at the regional level. These include the United Nations' Food and Agriculture Organization's Climate Change Adaptation in the Eastern Caribbean Fisheries Sector project, which seeks to increase resilience and reduce vulnerability to climate change through fisheries adaptation measures and capacity‐building for the fisheries sector (McConney, Cox, and Parsram, 2015; Turner, McConney, and Monnereau, 2020), and the Caribbean Catastrophe Risk Insurance Facility, creating, in 2019, the first parametric insurance for fishers in the neighbouring islands of Grenada and Saint Lucia (CCRIF SPC, 2019).

Set within this broad policy context, our findings build on a growing body of literature that emphasises the need to identify and place value on the more intangible social and relational components that matter for post‐disaster recovery or adapting to change (Gillam and Charles, 2018; Turner, McConney, and Monnereau, 2020). Often these critical dimensions of social organisation and cohesion, vital for community structure and capacity to adapt, rebuild, and foster resilience, are missed by large‐scale recovery, policy, or climate resilience initiatives. Recovery efforts in the aftermath of Hurricane Maria were facilitated by the activation of the Regional Response Mechanism coordinated by the Caribbean Disaster Emergency Management Agency with support from other international aid agencies, such as the Red Cross, Samaritan's Purse, and the World Bank. However, at present, CREAD is spearheading Dominica's efforts to improve future resilience through several key themes that emphasise strengthening communities, supporting them in absorbing stress through resistance or adaptation, and enhancing collective consciousness, to create the space to share experiences and the spiritual and cultural values that underpin how people live well, and how this can support ‘building back better’ (CoD, 2020b).

Using agency as an entry point to understand well‐being after Maria enabled this study to unpack the main elements in building resilience at the individual and community scale. People utilised multiple income streams and livelihoods, demonstrating flexibility in finding new and alternative opportunities. In addition, they drew on significant personal and social resources and the strength of relationships (pre‐existing local social networks) and personal resilience (coping and processing trauma) to recover from the devastating impacts. The fact that people consistently reference social relationships demonstrates that these are not only critical for well‐being, but also for recovery—as supported by research showing the importance of social networks for recovery after hazards (see, for example, Béné et al., 2016; Marin, 2019).

Some of the strategies pursued may have undermined people's well‐being and negatively affected resilience, such as the unintended livelihood implications of the NEP. Furthermore, well‐meaning government and external initiatives can also undermine well‐being. For instance, the donation by a charitable organisation called ‘Food for the Poor’ of a replacement boat with the name of the organisation on the side may have served to remind people that they were viewed as victims by others in more privileged positions. Scholarship in the fields of development and poverty studies has critiqued the categorisation of ‘poor’ as one that may not be salient for individuals (see, for example, Horner, 2020); rather, individuals may use categories such as gender, age, and national identity, whereas ‘poor’ is a socioeconomic position ascribed to them by ‘powerful’ others (Lister, 2004). People in these communities commonly spoke about values associated with independence, community cohesion, and reciprocal giving of time and goods in the recovery process, still ongoing to this day; not once did they apply negative socioeconomic connotations or label themselves as passive victims. Our analysis reveals that people are active agents, exercising their agency via multiple strategies to pursue recovery in line with well‐being goals; the external messaging of more powerful actors may negatively affect individual pride and self‐esteem.

Local social support networks were crucial in the immediate and medium‐term response to Maria; people moving away has the potential to disrupt them and the structural functioning of the community. While young people leaving rural areas for the reason of employment is not a new phenomenon (see, for example, Glendinning et al., 2003; Trimble and Johnson, 2013; Nandi and Nedumaran, 2021), the aspirations reported by community members illustrate that many entail young people staying and working within the community to build better lives for everyone—this perspective is also evident in other rural areas (see, for example, Giuliani et al., 2017). Some of the NEP placements actively undermined relational well‐being, as well as physical health and material wealth. The fact that some people who have left to take up employment still need assistance from their families in rural areas runs counter to the value people placed on independence for well‐being. This movement of people further negates aspects of relational well‐being and the resilience of those who remain in the villages.

Some participants' experiences of and views on the compensation process post Maria likely strengthened the value placed on independence and reliance on local social networks, as well as these actions demonstrating low levels of resistance to those in more powerful positions (such as the government). In addition to the social networks, individuals embedded in the community who work for local change or community benefits, also known as social entrepreneurs, play important roles in recovery efforts (Rayamajhee, Storr, and Bohara, 2022). The individual examples in this case, such as fishers travelling to neighbouring islands for food aid, and the key part they assumed in local recovery efforts highlight the importance of understanding local communities and facilitating bottom‐up recovery efforts that utilise the existing strengths of communities—a core theme in CREAD's mission (CoD, 2020b).

‘Building back better’ involves multiple forms of agency exercised simultaneously over the short, medium, and long term. While the more personal/everyday forms of agency focused on coping in the immediate aftermath of the hazard event, no form of agency was exercised in isolation. ‘Getting out’ and ‘getting organised’ represent longer‐term responses, and ‘getting (back) at’ represents an important context for the future design of ‘building back better’ policies and support. Understanding the interaction between all forms of agency and well‐being is vital context for resilience. Furthermore, comprehending this context in which people exercise their agency, and the trade‐offs they may make between well‐being and resilience, are important to consider in a policy aimed at enhancing resilience to hazards. While criticisms of resilience underscore the challenges in answering the question of ‘resilience of whom and to what?’ and how resilience does not always capture social dynamics (see, for example, Brown and Westaway, 2011; Cote and Nightingale, 2012; Béné et al., 2014), the approach used here has attempted to highlight these challenges and show how explicitly including an agency lens can offer an entry point to unpacking the diversity of livelihood strategies, decisions, and choices pursued by individuals, families, and communities in recovering after a disaster. Understanding this diversity is crucial for comprehending resilience, in its fullest sense—taking account of the material aspects of people's lives and livelihoods, as well as the relational or subjective dimensions of well‐being to improve the design of emergency response and recovery programmes.

## Conclusion

Hurricane Maria marked a turning point for many people: it was a hugely significant event for the people, communities, and nation of Dominica. A focus on macroeconomic recovery indicators is a critical component of post‐disaster recovery, as highlighted in Dominica's extensive PDNA (CoD, 2017) and the expansion of national social protection initiatives that provided additional employment to those in need (Beazley, 2018). However, our study reveals that while these efforts may fit with goals for national‐level resilience, they have the capacity to undermine individual and community (local) well‐being and resilience by influencing nuanced responses of agency (Lister, 2004). A key example is the NEP, which met the short‐term emergency response objective of the provision of a necessary safety net for many hundreds of people after Maria. However, our study exposes how this created disincentives for people to return to previous livelihoods, such as fishing or farming, with subsequent consequences for food security. Such trade‐offs between the immediate recovery response had implications for longer‐term recovery. The ability to ‘build back better’ is greatly diminished while people are locked in to a social protection programme that disincentivises a return to previous livelihoods. Furthermore, a lack of other support, such as insurance schemes for fishers to support building back when government compensation programmes are limited in terms of the amount of compensation or what is perceived as unequal or opaque decision‐making processes, may have contributed to changing livelihoods or people abandoning them.

The strategies evidenced in this study, including the flexibility of livelihoods that shift between different income earning options, informal transboundary food aid, the employment of multiple response strategies, and the vital role of collective community action are important to document. Using agency and well‐being to understand the motivations and context behind these responses provides insights into recovery processes and the ways in which resilience at different scales plays out. This is crucial for ‘building back better’ policies that aim to support recovery and promote well‐being yet involve trade‐offs at different levels. While these are not always straightforward to address or reconcile, applying conceptual frameworks that help to elucidate these complex social factors is a critical first step.

## Acknowledgements

We are grateful to the participants from the fishing communities and national institutions of the Commonwealth of Dominica who gave up their time to participate in the research. We also greatly appreciate the support of Dr Patrick McConney and his advice during the planning stages, and Dominica's Fisheries Division, which offered invaluable support before and during our visit. We would like to thank, too, Steve Joseph for his support prior to, during, and after the fieldwork, and for his constructive comments on the manuscript. Warm thanks are also extended to Dr Felipe de Jesus Gonzalez for his map‐making skills, and the three anonymous peer reviewers for their constructive comments that improved the paper. The work was funded by a Global Challenges Research Fund (quality‐related) grant awarded by the University of East Anglia in the United Kingdom (code: DEV31GFJF).

## Data availability statement

The data that support the findings of this study are available on request from the corresponding author. The data are not publicly available due to privacy or ethical restrictions.

## Author contribution statement

**Johanna Forster**: conceptualisation, methodology, data collection, analysis, writing—original draft, writing—review and editing, funding acquisition.

**Clare Shelton**: conceptualisation, methodology, analysis, writing—original draft, writing—review and editing, funding acquisition.

**Carole S. White**: conceptualisation, methodology, data collection, analysis, writing—review and editing, funding acquisition.

**Agathe Dupeyron**: analysis, writing—review and editing.

**Alena Mizinova**: analysis.

## Endnotes

2 Personal Communication with Steve M. Joseph, District Development Officer, Department of Local Government and Community Development, Government of Dominica, 2 February 2022.

3 Personal Communication with Steve M. Joseph, District Development Officer, Department of Local Government and Community Development, Government of Dominica, 2 February 2022.

4 The NEP is a training programme that targets youths aged between 18 and 25 years, offering them internships, community employment, and education mentorships. Internships focus on graduates working with government or private employers, and community employment centres on three days of work per week beautifying communities, carrying out roadside maintenance, or working in tourism. At least 2,000 people have been employed in the scheme annually since 2016 (Beazley, 2018; Carlisle, 2021).
